# Gastrointestinal Symptoms in Autism Spectrum Disorder: A Case-Control Study

**DOI:** 10.7759/cureus.68292

**Published:** 2024-08-31

**Authors:** Khaled Alqoaer, Maryam O Alenzi, Doha Abuharfel, Aziza Alenazi

**Affiliations:** 1 Pediatric Gastroenterology, King Salman Armed Forces Hospital, Tabuk, SAU; 2 Pediatric Medicine, King Salman Armed Forces Hospital, Tabuk, SAU; 3 Family Medicine, King Salman Armed Forces Hospital, Tabuk, SAU

**Keywords:** pediatric development, gastrointestinal, autism and gut, autism spectrum disorder (asd), autism and gastrointestinal dysfunction

## Abstract

Background and objectives

This study aimed to explore the frequency of gastrointestinal (GI) symptoms and associated risk factors among children with autism spectrum disorder (ASD).

Methods

This was a retrospective case-control study including children aged 2-14 years diagnosed with ASD by the Diagnostic and Statistical Manual of Mental Disorders (DSM)-5 criteria and the assessment card in the ASD center at King Salman Armed Forces Hospital. Data were obtained using a self-developed questionnaire that included demographic features, nutritional and behavioral characteristics, and GI symptoms in the previous six months. The control group consisted of typically developing (TD) children matched to the ASD group for age and gender. Syndromic autism with defined somatic abnormalities and recognized genetic causes (e.g., fragile X syndrome, tuberous sclerosis) were excluded

Results

A total of 146 ASD children and 114 normal children were included. No significant demographic differences were found between the groups. The ASD group had a higher frequency of low income and a significantly lower rate of exclusive breastfeeding in the first six months. GI symptoms, specifically constipation, abdominal gases and distension, diarrhea, undigested food particles in stool, and mouth ulcers, were significantly more frequent in the ASD group. Weight abnormalities (both increase and decrease) were also more common. Family history of ASD was significantly more in ASD children with GI symptoms while low maternal education was more in those without.

Conclusion

This study reveals a high prevalence of GI symptoms in ASD children. Family history of ASD and maternal education may influence the GI symptoms reported in ASD children

## Introduction

Autism spectrum disorder (ASD) is a neurodevelopmental illness that often manifests in early childhood. It is characterized by difficulties with social communication and confined repetitive behaviors [1]. ASD is associated with a higher mortality rate than the general population by a factor of 2-10, making it a serious issue despite its frequency [2]. Globally, the prevalence of ASD has increased dramatically over the past 40 years, rising 20-30 times to an alarming one in 132 people [3-5]. In the Arabian Peninsula, prevalence rates vary from 1.4 to 185 per 10,000 [6]. The increasing frequency of ASD underscores the need for thorough investigation and effective intervention techniques to address its expanding impact on individuals and communities.

Over 90% of children with ASD have at least one co-occurring medical issue [7], with GI disorders being the most common, in addition to intellectual impairment, movement abnormalities, and sleep difficulties [8-9]. GI issues are notable non-neurological symptoms of ASD, linked to more severe abnormalities in critical ASD domains such as behavior and cognition [10-13]. Due to sample characteristics and methodology variations, the prevalence of GI symptoms in ASD ranges from 9% to 91% [14]. However, the precise mechanisms behind the high frequency of GI symptoms in ASD are unknown. GI symptoms are more prevalent in children with ASD, with constipation, diarrhea, and stomach pain being the most common [15]. This finding emphasizes the importance of understanding and treating GI issues in ASD. Prior research has shown substantial variance in the prevalence of GI symptoms among persons with ASD, highlighting the need for regional studies to understand the condition’s global distribution better.

## Materials and methods

A retrospective case-control study was conducted at the Prince Mohammed bin Salman Centre for Autism and Development Disorder, King Salman Armed Forces Hospital, in Tabuk, Saudi Arabia. The study included individuals aged 2-14 years diagnosed with ASD based on DSM-5 criteria. Children with syndromic autism, defined somatic abnormalities, or recognized genetic causes (e.g., fragile X syndrome, tuberous sclerosis) were excluded. The study was approved by the Research Ethical Team at King Salman Armed Forces Hospital, Tabuk (approval number: KSAFH-RET-2024-588).

The sample size was determined using the standard formula: N = Z(2)PQ/D(2), where N is the sample size, P is the probability of success, Z is the standardized variable corresponding to confidence level, D is the desired marginal error, and Q is (1-P). Here, we took P=50%, D=10%, Z=95%. A sample size of at least 100 participants in each group was found adequate to run the study. A total of 146 children with ASD and 114 controls (healthy children without ASD) were thus included.

Data were collected through a self-developed questionnaire, which included demographic details, parents' education (low education if up to high school or less), and family income based on Saudi Arabian standards. Also, the data included some behavioral characteristics like sleep problems, self-mutilation or violent behavior toward others, and repetitive behavior. The participants were screened for specific GI symptoms experienced in the previous six months as reported by parents or caregivers. The symptoms included mouth ulcer, dysphagia, vomiting, abdominal gases, nausea, abdominal distension, abdominal pain, diarrhea, constipation, bad smell stool, food particles in stool, increased weight or weight loss, abnormal appetite, selective eating, and food allergy. The control group consisted of TD children matched for age and gender, recruited from well-baby and school health clinics.

Statistical analysis

Descriptive statistics were used to summarize demographic and clinical characteristics. The chi-square test assessed associations and differences between categorical variables, while the independent samples t-test compared means between groups for continuous variables. Fisher’s exact test was used for variables with small sample sizes. Analysis of variance (ANOVA) was considered for comparing means across multiple groups. Statistical analysis was performed using IBM SPSS Statistics for Windows, Version 24.0 (Released 2016; IBM Corp., Armonk, New York, United States) with a significance level of p < 0.05.

## Results

Table 1 compares various demographic and clinical variables between individuals with ASD and the control group. The groups were matched for age and gender. A significant difference was observed in the rate of low income among ASD families (45.1% vs. 18.7%, p < 0.001). The rate of exclusive breastfeeding during the first six months was lower in the ASD group (17.9% vs. 30.7%, p < 0.016). A significant difference was noted in the family history of GI complaints (40% vs. 60.2%, p < 0.002). The mean age at weaning was significantly higher in the ASD group (7.5±3.21 vs. 6.57±2.61 months, p < 0.011).

**Table 1:** Demographic and clinical variables between ASD and control groups ASD: autism spectrum disorder; SVD: spontaneous vaginal delivery

Variables	ASD Group (n=146)	Control Group (n=114)	P-value
Gender, male, n (%)	105 (71.9%)	81 (71.1%)	0.878
Age in years, mean±SD	7.79±3.01	7.59±2.58	0.563
Mother's age in years, mean±SD	37.66±6.88	36.56±6.44	0.196
Father's age in years, mean±SD	42.48±7.73	42.71±8.89	0.831
Mode of delivery, SVD, n (%)	102 (70.8%)	88 (78.6%)	0.160
Family history of ASD, n (%)	22 (15.8%)	11 (9.7%)	0.154
Father's low education, n (%)	93 (64.6%)	66 (57.9%)	0.273
Mother's low education, n (%)	84 (58.3%)	58 (50.9%)	0.232
Low income, n (%)	65 (45.1%)	14 (18.7%)	0.005*
Exclusive breastfeeding for first six months, n (%)	26 (17.9%)	35 (30.7%)	0.016*
Breastfeeding duration in months, mean±SD	9.12±9.52	8.52±7.71	0.611
Age at weaning in months, mean±SD	7.5±3.21	6.57±2.61	0.011*

Table 2 highlights behavioral challenges associated with ASD. Significant and expected differences were found in sleep problems, self-mutilation, violent behavior toward others, and obsessive repetitive behavior between the ASD and control groups.

**Table 2:** Some behavioral characteristics of the ASD and the control groups ASD: autism spectrum disorder

Variables	ASD Group, n (%)	Control Group, n (%)	P-value
Sleep Problem	51 (34.9)	15 (13.2)	0.000
Self-Mutilation	48 (32.9)	4 (3.5)	0.000
Violent Behavior Toward Others	56 (38.4)	18 (15.9)	0.000
Obsessive Repetitive Behavior	40 (27.8)	2 (1.8)	0.000

The overall prevalence of any GI symptoms in the ASD group was 82% compared to 62% in the control group (p < 0.001). Table 3 compares various GI symptoms between both groups, showing a significantly higher prevalence of most GI symptoms in the ASD group namely mouth ulcers, abdominal gases, abdominal distension, constipation, diarrhea, weight abnormalities, abnormal appetite, and selective eating in the ASD group. No significant difference was found in vomiting, nausea dysphagia, abdominal pain, and food allergy. The ASD group had a significantly higher mean number of GI symptoms (2.83±2.45 vs 1.92±2.14, p < 0.002).

**Table 3:** Gastrointestinal symptoms comparison between ASD and control groups Data given as n (%) except for in Number of GI Symptoms, which is given as mean±SD. ASD: autism spectrum disorder

Variable No (%)	ASD Group, n (%)	Control Group, n (%)	P-value
Mouth ulcer	26 (18.10%)	10 (8.80%)	0.033
Dysphagia	17 (11.80%)	13 (11.40%)	0.92
Vomiting	20 (13.80%)	22 (19.30%)	0.233
Abdominal gases	69 (47.90%)	36 (31.60%)	0.008
Nausea	27 (18.80%)	18 (15.80%)	0.534
Abdominal distension	42 (29.20%)	13 (11.40%)	0.001
Abdominal pain	59 (41.00%)	41 (36.00%)	0.412
Diarrhea	42 (29.20%)	21 (18.40%)	0.046
Constipation	61 (41.80%)	27 (23.70%)	0.002
Bad smell stool	74 (50.70%)	16 (14.00%)	0.000
Food particles in stool	37 (25.50%)	4 (3.50%)	0.000
Increase weight	31 (21.4%)	12 (10.5%)	0.020
Weight loss	44 (31.7%)	16 (14%)	0.001
Abnormal appetite	66 (45.5%)	7 (6.1%)	0.000
Selective eating	103 (71%)	43 (37.7%)	0.000
Food allergy	20 (13.8%)	12 (10.5%)	0.42
Number of GI symptoms, mean±SD	2.83±2.45	1.92±2.14	0.002

Table 4 investigates the differences in sociodemographic and behavioral factors among the ASD groups with and without GI symptoms. No statistically significant differences were found regarding age, gender, breastfeeding duration, age at weaning, mode of delivery, or parental education and income. An important difference was observed in the family history of ASD and other mutilation. Furthermore, a significant difference was also found in maternal low education which was more in the ASD group without GI symptoms (p = 0.046).

**Table 4:** Gastrointestinal symptoms in ASD children without and with GI symptoms ASD: autism spectrum disorder; GI: gastrointestinal

Variable	ASD without GI Symptoms (n=28)	ASD with GI Symptoms (n=118)	P-value
Gender (male), n (%)	18 (64.3)	87 (73.7)	0.318
Age (years), mean±SD	7.5±2.59	7.83±3.10	0.705
Breast Feeding Duration, mean	9.10	9.12	0.989
Age at Weaning, mean±SD	8.57±3.54	7.26±3.10	0.089
Mode of Delivery SVD, n (%)	17 (63)	85 (72)	0.318
Exclusive Breast Feeding for First Six Months, n (%)	5 (18.5)	21 (17.8)	0.93
Father's Low Education, n (%)	18 (64.3)	75 (64.7)	0.971
Mother's Low Education, n (%)	21 (75)	63 (54.3)	0.046
Family History of ASD, n (%)	1 (3.60)	21 (18.9)	0.047
Family History of GI Disorder, n (%)	6 (24)	46 (43.80)	0.069
Family Low Income, n (%)	9 (32.1)	56 (48.30)	0.124
Sleep Problem, n (%)	6 (21.4)	45 (38.1)	0.96
Self-mutilation, n (%)	5 (17.9)	43 (36.4)	0.06
Violent Behavior Toward Others, n (%)	6 (21.4)	50 (42.4)	0.04
Repetitive Behavior, n (%)	6 (21.4)	34 (29.30)	0.403

## Discussion

Our results indicate a higher frequency of GI symptoms among children with ASD compared to the control group. Various studies have consistently shown higher rates of GI symptoms in individuals with ASD than in the general population. A meta-analysis by McElhanon et al., which reviewed 15 studies, found that children with ASD are more than four times as likely to experience GI symptoms as their typically developing peers [15]. The prevalence of any GI symptom in individuals with ASD ranged from 23% to 70%. Another study by Chaidez et al. reported that 49% of children with ASD had at least one GI symptom [16]. Lefter et al. noted an 83% prevalence of GI symptoms in ASD children, with diarrhea, constipation, and abdominal pain being the most common complaints [17].

In our study, we found that ASD children reported significantly higher rates of mouth ulcers, abdominal gases, abdominal distension, constipation, diarrhea, weight fluctuations, abnormal appetite, food selectivity, bad-smelling stool, and abnormal appetite compared to the control group. Additionally, the ASD group exhibited a significantly lower rate of exclusive breastfeeding in the first six months and an older age at weaning. This raises questions about the potential role of breastfeeding and nutritional practices in the development and pathogenesis of ASD, as many studies have suggested a protective effect of breastfeeding against the development of ASD [18-22].

Our findings also highlight socioeconomic factors, with a higher frequency of low income among ASD families, which may add additional challenges for these families. Comparing ASD children with GI symptoms to those without, we observed a significant difference regarding the family history of ASD and other mutilation behaviors. The family history of ASD may indicate a genetic factor or predisposition leading to more severe multisystem involvement of ASD. In 2014, Chaidez et al also noted that ASD children with frequent abdominal pain, gaseousness, diarrhea, constipation, or pain during stooling scored worse on measures of irritability, social withdrawal, stereotypy, and hyperactivity compared to children without frequent GI symptoms [16]. GI symptoms were associated with increased severity of behavioral problems in children with ASD. According to Buie et al., a panel of experts noted that GI disorders are common in individuals with ASD and can significantly affect their behavior and quality of life, emphasizing the need for thorough evaluation and treatment of GI issues in this population [23]. Another interesting finding is the lower rate of low maternal education in the group without GI symptoms. While there is no clear explanation, we speculate that less educated mothers may have less awareness of GI symptoms, leading to under-recognition of the issue. Previous studies have shown similar correlations between maternal education level and the likelihood of an autism diagnosis [24].

This study has a few limitations. Recall bias cannot be ignored due to the retrospective nature of the data collection. Additionally, we aimed to provide a general overview of GI involvement in ASD, so we did not delve deeply into the specifics of these symptoms. However, to our knowledge, this is the first study from Saudi Arabia and the Middle East to address this issue. Further research is needed to explore the relationship between ASD and the GI system in more detail

## Conclusions

This study highlights the significant frequency of GI symptoms in individuals with ASD and underscores the importance of addressing these symptoms in clinical practice. Many individuals with ASD experience a range of digestive issues. Ongoing monitoring and further research are crucial for improving diagnosis, intervention, and tailored healthcare strategies for ASD patients with GI concerns.
